# The effects of neck exercise in comparison to passive or no intervention on quantitative sensory testing measurements in adults with chronic neck pain: A systematic review

**DOI:** 10.1371/journal.pone.0303166

**Published:** 2024-05-03

**Authors:** Daniel Osborne, Ferozkhan Jadhakhan, Deborah Falla

**Affiliations:** 1 Centre of Precision Rehabilitation for Spinal Pain (CPR Spine), School of Sport, Exercise and Rehabilitation Sciences, College of Life and Environmental Sciences, University of Birmingham, Edgbaston, Birmingham, United Kingdom; 2 Faculty of Health, Education and Life Sciences, City South Campus, Birmingham City University, Birmingham, United Kingdom; University of Rome, ITALY

## Abstract

**Background:**

Previous systematic reviews have identified the benefits of exercise for chronic neck pain on subjective reports of pain, but not with objective measures such as quantitative sensory testing (QST). A systematic review was conducted to identify the effects of neck specific exercise on QST measures in adults with chronic neck pain to synthesise existing literature and provide clinical recommendations.

**Methods:**

The study protocol was registered prospectively with PROSPERO (PROSPERO CRD42021297383). For both randomised and non-randomised trials, the following databases and trial registries were searched: AMED, CINAHL, Embase, Google Scholar, Medline, PEDro, PubMed, Scopus, SPORTDiscus, Science Citation Index and Social Science Citation Index from Web of Science Core Collection, clinicaltrials.gov, GreyOpen, and ISRCTN registry. These searches were conducted from inception to February 2022 and were updated until September 2023. Reference lists of eligible studies were screened. Study selection was performed independently by two reviewers, with data extraction and quality appraisal completed by one reviewer and independently ratified by a second reviewer. Due to high heterogeneity, narrative synthesis was performed with results grouped by exercise type.

**Findings:**

Three trials were included. Risk of bias was rated as moderate and the certainty of evidence as low or moderate for all studies. All exercise groups demonstrated statistically significant improvement at an intermediate-term follow-up, with progressive resistance training combined with graded physical training demonstrating the highest certainty of evidence. Fixed resistance training demonstrated statistically significant improvement in QST measures at a short-term assessment.

**Interpretation:**

Fixed resistance training is effective for short-term changes in pain sensitivity based on low-quality evidence, whilst moderate-quality evidence supports progressive resistance training combined with graded physical training for intermediate-term changes in pain sensitivity.

## Introduction

Neck pain is a common musculoskeletal disorder regularly causing considerable pain, disability, socioeconomic cost and decreased psychosocial wellbeing [1–3]. It is the second most prevalent musculoskeletal condition and the fourth leading cause of disability worldwide [3, 4], with a global age-standardised point prevalence of 3551.1 per 100,000 individuals and an incidence rate of 806.6 per 100,000, with the largest increases in point prevalence over the past three decades noted in the UK [2]. Prevalence peaks during the fifth and sixth decades of life, and the most recent available data indicate 288.7 million global cases in 2017 [2]. Some 70% of adults may experience neck pain during their lifetime [5, 6]. Whilst non-specific neck pain (NSNP) often resolves within a few months, approximately 50% of cases develop into chronic neck pain [6].

Neck pain is commonly diagnosed as ‘non-specific’ due to the multiplicity of causes and difficulty in distinguishing clear underlying pathology [7], with various mechanical factors, including poor motor control, strength deficits and previous cervical injury all associated with the condition [6,8]. Numerous risk factors including previous spinal pain/trauma, poor posture, decreased psychological wellbeing and poor levels of social support, likely perpetuate its presence and worsen prognosis [6, 9–11].

Clinical guidelines advocate multimodal management, including manual therapy, advice and education, ergonomic/lifestyle modification, pharmacological intervention and exercise for the management of chronic neck pain [6]. Exercise for neck pain includes approaches such as motor control, strengthening, proprioceptive or endurance training [6] which are known to stimulate neuromuscular adaptations to training in addition to improvements in pain and function [12–14]. Clinical guidelines [6] do not currently recommend one specific form of exercise over another for the management of chronic neck pain.

Previous systematic reviews have quantified the benefits of exercise for neck pain [15, 16] by assessing the effects of exercise on subjective reports of pain (e.g. visual analogue scale to rate a change in neck pain intensity) and disability (e.g. Neck Disability Index). In contrast, no systematic review has evaluated the current evidence on whether exercise for neck pain leads to a change in more objective measures of pain sensitivity assessed with quantitative sensory testing (QST).

QST is a psychophysical outcome measure, utilising different modalities, to quantifiably assess a patient’s somatosensory response to specific stimuli [17, 18], and is increasingly utilised for providing an objective marker for the assessment of central and peripheral pain in different musculoskeletal conditions. QST typically consists of various mechanical, thermal, electrical or chemical stimuli and may include static measures, such as pain pressure thresholds (PPTs) and thermal pain thresholds, or dynamic measures, such as temporal summation or conditioned pain modulation [19, 20]. Previous systematic reviews considered the effects of exercise measured by QST for shoulder pain [18] and manual therapy and exercise for temporomandibular disorders [21], both demonstrating benefit.

Whilst the subjective concept of pain is typically assessed using patient reported outcome measures in clinical practice, the use of QST within a research setting allows to develop a quantifiable understanding of pain response as an output in relation to a specific stimulus or input, which can create improved understanding of patient response under experimental conditions. This offers further insight into factors that influence pain, including pain mechanisms, sensory response of fibre types, and potentially could allow an improved prediction of treatment efficacy and the analgesic benefit of treatments, which can subsequently be translated into clinical practice.

This review aims to synthesise the existing body of primary evidence and identify whether neck specific exercise (i.e., exercise of the neck muscles as opposed to general physical activity) is effective for a reduction of pain sensitivity measured via QST in adults with chronic neck pain. Furthermore, this review aims to determine whether one type of exercise demonstrates greater improvement in comparison to a control intervention to provide early indications of whether one type of exercise is superior to another. Given the current lack of synthesised evidence and clinical guidance as to the best type of exercise for chronic neck pain, in synthesising the current evidence, this review aims to assist in influencing clinical practice by informing practitioners of best evidence-based modalities of the treatment for chronic neck pain. We considered exercise protocols ≥2 weeks so as to determine the effects of an exercise programme, rather than a single bout of exercise.

## Materials and methods

### Protocol and registration

This review was conducted and is reported following the Preferred Reporting Items for Systematic Reviews (PRISMA) guidelines [22, 23] with a pre-defined protocol registered on the PROSPERO database (CRD42021297383). Deviations from original protocol included a slight delay in completion of literature searching, with completion in February 2022 as opposed to January 2022. The search was updated and reviewed once more up until September 2023. Independent secondary reviewer completion of data extraction and risk of bias assessment was adjusted and independently completed by one reviewer (DO) and then independently ratified by a second reviewer (FJ). The second reviewer was changed (to FJ) due to availability. These deviations from protocol were made due to reviewer availability and review timeframe. Changes of the secondary reviewer were made prior to the commencement of the review, thus reducing the risk of bias and inconsistency in the review process.

### Selection criteria

The eligibility criteria for this review were developed in line with PICOS (Population, Intervention, Comparator, Outcome, Study Design) to ensure appropriate consideration was applied for important domains [24, 25] with scoping searches and consultation of previous studies with similarities in methodology [15, 16, 18, 21, 26] and Cochrane Back and Neck Group Guidelines [27] to help develop eligibility criteria. Development of eligibility criteria was also assisted by two reviewers with expertise in the field and in systematic review methodology (DF/FJ). Trials not written in English and animal studies were excluded from this review.

### Population

Criteria included individuals aged ≥18 years old with chronic (≥3 months) neck pain, including chronic non-specific neck pain, and pain experienced as a result of a whiplash injury [27]. Exclusion criteria included any individuals with specific pathology, such as cervical radiculopathy, disc herniation, infection, inflammatory arthropathies, previous spinal surgery, spinal stenosis and primary headaches such as migraine or tension type headache, due to somatosensory profile variances experienced by some patient populations with specific pathology when assessed by QST [28].

### Intervention

Any form of neck-specific exercise, such as motor control, strengthening or stretching exercise, performed for a minimum of two weeks with no additional treatment other than advice and education (including but not limited to, educational literature and clinician advice) was considered for inclusion in this review. Any study comparing two or more types of neck-specific exercise interventions was also considered with the effects of each exercise intervention assessed through separate groupings.

### Comparator

Any study including no intervention or passive interventions (e.g., advice and education or manual therapy) as the comparator was considered for inclusion, to ensure that the effects of exercise could be appropriately assessed without the risk of cofounding variables, resulting from active training modalities, affecting the results of comparator groups [29]. Manual therapy refers to any passively applied movement technique by a practitioner such as spinal mobilisation or manipulation.

### Outcome measures

As per previous studies assessing QST on different anatomical locations to this study [21, 18], outcome measures of interest included any QST modalities. Examples of these modalities include, but are not limited to, thermal, electrical, chemical and/or mechanical stimuli applied to the joint, muscle or skin and/or studies describing stimulus site, pain intensity and modality. Examples of these modalities include, but are not limited to, PPTs, thermal pain thresholds, conditioned pain modulation and temporal summation. With the focus of this review towards neck pain, QST sites local to the cervical spine were considered, with studies only assessing remote QST sites excluded.

### Study design and setting

Any randomised-controlled trial and/or non-randomised study (including but not limited to quasi-experimental studies, cohort studies, controlled before-and-after studies) of a neck-specific exercise intervention was considered for inclusion in this review. Any setting, such as primary or secondary care, or laboratory-based was considered. Studies were not restricted by geographical location.

### Information sources

The following databases were searched: AMED, CINAHL, Embase, Medline, PEDro, PubMed, Scopus, SPORTDiscus, and Science Citation Index and Social Science Citation Index from Web of Science Core Collection. Additionally, Google Scholar was searched. Electronic databases were searched from inception through February 2022 and updated in September 2023. Unpublished literature, ongoing trials and other grey literature were searched using the following databases and trial registries: clinicaltrials.gov, OpenGrey (up until 1^st^ December 2020 as it was discontinued after this time) and ISRCTN registry. Citation searching was performed by reviewing reference lists from eligible studies to identify any further articles that may not have been identified from database searching.

### Search strategy

The search strategy was developed with guidance from the author team with expertise in the methodology of systematic reviews (DF, FJ). Searches were completed independently by a single reviewer (DO). All data relating to the search were imported into and stored, with duplicates removed, using EndNote Version 20 software [30]. The search strategy for all databases and search engines is presented in S1 File.

### Selection process

Two reviewers (DO/FJ) independently retrieved and assessed articles following the implementation and completion of the search strategy. To ensure that the most recent articles are included in the review, this procedure was repeated after the original search strategy. Articles were screened by title, abstract and full text in order to apply the eligibility criteria. Whilst EndNote Version 20 software [30] was used to remove duplicate articles, no automated software was used throughout the selection process. Consensus was reached between the two reviewers regarding articles meeting the eligibility criteria and subsequent inclusion within this review. A third reviewer with subject and methodological expertise (DF) was available for consultation if needed. No corresponding author contact was required to obtain full-text reports or clarify uncertainty during the selection process.

### Data collection process

Data were independently extracted by one reviewer (DO) using pre-defined data extraction tables. Data extraction was independently ratified by a second reviewer (FJ). Data were extracted for the following items: study design, location and setting; participant age, gender, ethnicity and additional characteristics presented in primary articles; sample size, length of follow-up, outcomes of interest (primarily QST modalities), intervention and comparator type; and items related to risk of bias, methods and results of statistical analysis. Follow-up durations were termed as short-term (<3 months), intermediate-term (3 months to <12 months) and long-term (≥12 months) [26]. All data were extracted manually, with no requirement to utilise software tools. There were no missing data from any of the included studies. However, some studies did not report all raw data. It was not possible to retrieve raw data from the authors of the studies included within this review; effort was made to contact authors at least twice by email. Completed data extraction forms for all studies included within this review are presented in S2 File.

### Risk of bias

The Cochrane Risk of Bias 2 (RoB-2) tool [31] was used to assess internal validity and bias across the studies included in this systematic review. RoB-2 is a tool that assesses the risk of bias in a study from five domains relating to the ‘randomisation process’, ‘deviation from intended intervention’, ‘missing outcome data’, ‘measurement of the outcome’ and ‘selection of the reported result’. Bias in each domain is rated as either ‘low risk’, ‘some concerns’ (moderate risk) or ‘high risk’.

Whilst there is currently a lack of available data regarding the validity and reliability of the RoB-2 tool for assessing randomised-controlled trials, the tool was selected due to its ability to thoroughly assess internal validity through consideration of multiple sources of bias across the five domains [32]. Further, as opposed to similar tools, the RoB-2 tool provides an overall estimation of risk of bias, as well as individual domain consideration, allowing for a transparent understanding of all sources of potential bias and its overall effect on a study [33].

As difficulty has been noted in the interpretation of different domains and the application of the tool to primary research, especially for non-experts in subject matter [34], following independent risk of bias assessment by a single reviewer (DO), a second reviewer (FJ) independently ratified the results of the RoB-2 assessment to ensure accuracy and consistency of application. A third reviewer (DF) was available should any disagreements have occurred but was not required. Inter-rater reliability between assessors was not statistically assessed. The RoB-2 Cribsheet and Microsoft Excel assessment tool [31] were utilised to assess each individual domain and provide an overall estimation of risk of bias. The full report for the RoB-2 assessment is presented in S3 File.

### Certainty of evidence

The quality of evidence for each study was assessed using the Grading of Recommendations, Assessment, Development and Evaluation (GRADE) assessment tool and GRADEpro software [35–38]. GRADE is a commonly used tool for the assessment and quality rating of evidence [39] that enables reviewers to make transparent, evidence-based decisions regarding the quality of evidence of healthcare-based research [40] and is recommended for use by the Cochrane Handbook for Systematic Reviews of Interventions [41]. Decisions are formulated through the assessment of five domains: ‘risk of bias’, ‘inconsistency’, ‘indirectness’, ‘imprecision’ and ‘publication bias’, with each domain rated as either ‘very low’, ‘low’, ‘moderate’ or ‘high’ quality evidence and a total rating calculated to suggest an overall quality rating for a particular study [38].

One reviewer (DO) independently assessed all articles to attribute a quality of evidence rating to each. All studies were assessed independently as it was not possible to pool data due to heterogeneity across the three included studies. A second reviewer (FJ) independently ratified the results of the assessment and provided suggestions for the presentation of results. A third reviewer (DF) was available to settle any disagreement.

### Data analysis and synthesis methods

Due to a high level of heterogeneity between the included studies (e.g., gender of participants, baseline duration of pain, intervention characteristics and dosage and timing of primary outcome assessment), lack of reported data required and variability between the effect estimates utilised by the three included studies, a meta-analysis was not possible to pool overall effect estimates. It was also not possible to conduct statistical assessment for heterogeneity due to lack of required data. Because of this, narrative synthesis was undertaken and reported in line with synthesis without meta-analysis (SWiM) guidelines [42] and PRISMA guidelines [22, 23]. It is acknowledged that the absence of a meta-analysis may reduce the precision of direction of effect estimates and thus the accuracy and generalisability of results.

### Effect measures

As per SWiM guidance, *p*-values (where *p*<0.05 is considered statistically significant) were selected as the standardised metric for the primary outcome measure (for QST) as all studies consistently reported these data. It was not possible to combine *p-*values across studies, therefore, this standardised metric is reported individually for each study. Where reported, effect size was also presented in the narrative synthesis of results. Study characteristics and summaries of intervention are presented in tables within the results section of this report.

### Grouping of studies for synthesis

For the narrative synthesis of evidence, studies were grouped by exercise type. Although this review protocol initially planned for studies to be grouped by primary outcome measure, all studies utilised the same primary outcome measure, PPT, which would have resulted in only one grouping for all studies. Because of this, and due to variance between studies in exercise intervention protocol, it was deemed more appropriate to group studies by exercise intervention type to explore whether exercise type affected the primary outcome. Exercise types were progressive resistance training (PRT), PRT combined with graded physical training and fixed resistance training (FRT).

The direction of effect was calculated for all studies based on final follow-up assessment of PPTs and visually presented in a direction of effect plot. Calculation was guided by procedure presented by Boon and Thomson [43]. Vote counting was utilised to assess whether there was a positive impact of exercise intervention at intermediate-term follow-up, which is considered an appropriate method to be used in narrative synthesis [44]. Direction of effect was not calculated and vote counting not used at short-term assessments across studies due to variability in assessment timings and available data for each study resulting in inability to apply this technique. The findings for these time-points are presented individually as *p*-values and discussed.

## Results

### Study selection

**Fig 1:**
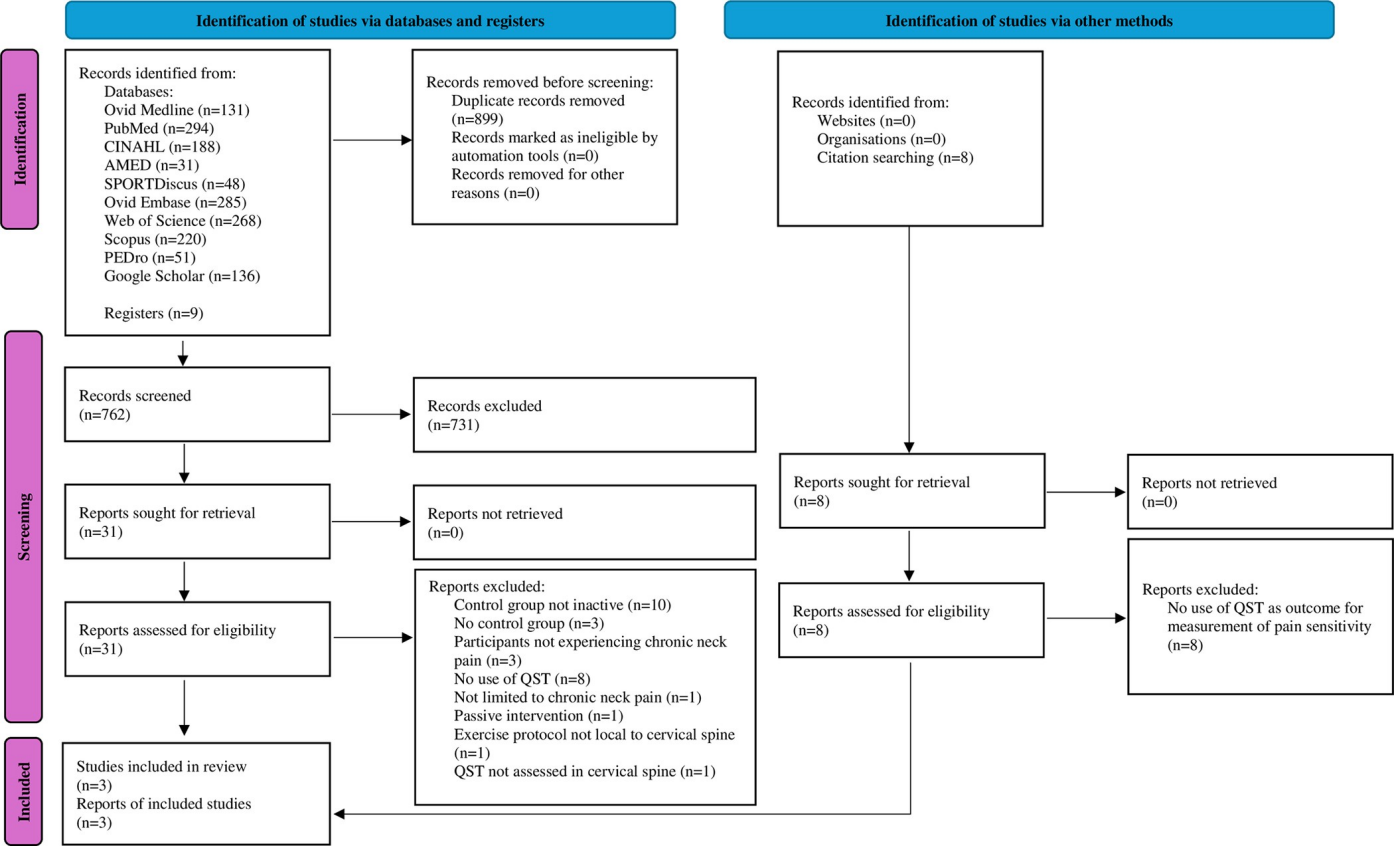
PRISMA flow diagram.

The systematic literature search for this study produced 1661 overall potentially relevant results from all databases searched, as presented in the PRISMA Flow Diagram [23], presented in Fig 1. Of the identified studies, 899 records were removed as duplicates, resulting in the screening of 762 records. 731 records were removed following title and abstract assessment in comparison to the eligibility criteria and 31 full-text records were sought for retrieval. Following full-text assessment in comparison to the eligibility criteria, three randomised-controlled trials met the eligibility criteria and were included for final synthesis of results. Citation screening also identified a further eight potential studies, all of which were excluded due to the lack of use of QST as an outcome measure for the assessment of pain sensitivity in each of the identified studies. A flow diagram of the study selection process is presented in Fig 1.

### Study characteristics

Data from each identified study were extracted using bespoke data extraction forms. A summary of characteristics for the three studies [45–47] is presented in Table 1. Studies included in this review were published between 2016 and 2020 in three separate countries; one in Spain [45], one in China [46], and one in Denmark [47]. One study adopted a multi-centre design [47], one single-centre [45] and one recruited from multiple places of employment [46]. Two studies utilised intention-to-treat analysis [46, 47] and one did not [45], with one study also using per-protocol analysis for the assessment of adherence [47].

**Table 1:** Summary of study characteristics.

Study and Design	Year	Country	Participants/Sample	Intervention (Duration)	Comparator	Follow-Up	Outcome (QST)	Key Findings	Exercise Modality
Bernal-Utrera et al [45] (RCT)	2020	Spain	Participants aged 18–50 years old, with current neck pain experienced for ≥ previous 12 weeks. Total sample size at baseline n = 69. (TE n = 23; MT n = 23; Control n = 23). Sample size at follow up n = 65 (TE n = 23[22% male; 78% female]; MT n = 22 [23% male; 77% female]; Control n = 20 [25% male; 75% female]). Total analysed due to per-protocol analysis n = 65. Excluding MT, total analysed n = 43. *NB No gender distribution data reported at baseline*. **Mean age (years):** Manual therapy group = 42.95 ± 2.89. Therapeutic exercise = 36.78 ± 2.89. Control = 36.90 ± 2.89. **Mean height (cm):** Manual therapy = 168.05±3.47, Therapeutic Exercise = 165.48±2.10, Control = 168.85 ±2.19. **Mean weight:** MT = 69.86±3.47, TE = 65.26 ± 2.35, Control = 70.45 ± 2.15. **Mean BMI (kg/m2):** MT = 24.67 ± 1.13, TE = 23.8 ± 0.72, Control = 24.75 ± 0.75.	PRT. Exercise performed once daily for 3 weeks (21 sessions).	Sham MT.	Measures assessed at baseline, week 2, week 4, week 12.	PPT	Statistically significant improvement noted between groups for TE in comparison to control at week 12 follow-up evaluation (*p* = 0.001). No significant differences were recorded between groups at weeks 2 (*p =* 0.991) or 4 (*p* = 0.415). TE demonstrated a medium effect size in comparison to control at 4 weeks (*R*^*2*^ = 0.082) and a large effect size at week 12 (*R*^*2*^ = 0.328). TE demonstrated a statistically significant intragroup improvement at 12-week follow-up (*p* = 0.001), but not at week 2 (*p* = 0.729) or week 4 (*p* = 0.061). Control group demonstrated no statistically significant change (*p>*0.05).	Protocol based on progressive load throughout three phases. Protocol performed once per day for a total of 21 sessions over a three-week period with each phase lasting one week. Patients were taught exercises during first session. Phase one = deep cervical flexor activation and recruitment. Phase two = deep and superficial cervical flexor co contraction isometric exercise. Phase 3 = eccentric recruitment of flexors and extensors. During each phase, the exercises from the preceding phase were also completed.
Li et al [46] (RCT)	2017	China	Female participants aged 20–55, who use computers daily and have experienced frequent computer related neck pain for >1 year and have experienced neck pain in the last 7 days. Total sample size at baseline n = 109. (PRT n = 38; FRT n = 35; Control n = 36). Sample size at follow-up n = 102 (PRT n = 36; FRT n = 32; Control n = 34). Total analysed due to intention-to-treat analysis n = 109. *NB 100% of participants were female*. **Mean Age (years):** Control = 34.1 ± 8.2. Progressive Resistance Training (PRT) = 35.6 ± 7.9. Fixed Resistance Training (FRT) = 33.7 ± 9.0. *p = 0*.*893 (between groups)*. **Mean height (cm):** PRT = 163.2 ± 7.5. FRT = 163.7 ± 6.2. Control = 165.8 ± 7.9. p = 0.269 **Mean weight (kg)**: PRT = 55.7 ± 8.4. FRT = 58.7 ± 9.6. Control = 59.6 ± 9.0. p = 0.380 **Mean BMI (kg/m2)**: PRT = 21.0 ± 3.7. FRT = 22.1 ± 4.20. Control = 21.9 ± 3.9. p = 0.521 **Mean pain duration (years):** PRT = 3.4 ± 1.9. FRT = 3.5 ± 2.2. Control = 3.8 ± 2.4. p = 0.796 **Mean job experience (years)**: PRT = 9.2 ± 5.8. FRT = 8.6 ± 6.1. Control = 9.7 ± 5.5. p = 0.593 **Mean working duration (days/week):** PRT = 5.3 ± 0.6. FRT = 5.5 ± 0.5. Control = 5.1 ± 0.4. p = 0.086 **Mean computer use (h/day):** PRT = 7.3 ± 2.5. FRT = 7.6 ± 2.3. Control = 7.0 ± 1.8. p = 0.325	PRT: exercise performed ≥3 times per week for 6 weeks. FRT: exercise performed ≥3 times per week for 6 weeks.	Advice and education including weekly discussion groups.	Measures assessed at baseline, week 2, week 4, week 6 and 3 months.	PPT	PRT demonstrated statistically significant improvements at weeks 2 (*p* = 0.017), 4 (*p*<0.001), 6 (*p*<0.001) and 3-month follow-up (*p*<0.001) in comparison to control. FRT demonstrated a significant improvement in comparison to control at weeks 4 (*p* = 0.001), 6 (*p*<0.001) and 3-month follow-up (*p*<0.001), but not at week 2 (*p* = 0.075). No statistically significant difference was noted between PRT and FRT groups at any time-point (*p>*0.05). Control group demonstrated no statistically significant change (*p>*0.05).	**PRT:** Performed four Theraband resisted isometric cervical exercises: flexion, extension, left and right lateral flexion, using a Theraband. Neck exercises were performed in sitting with the cervical spine and head in the neutral anatomical position. Training load progressed over 6 weeks to achieve progressive overload. Starting resistance = 30% of patient’s maximal strength. Resistance increased to 50% maximal strength at week 2, and 70% maximal strength at week 4. Hand-held dynamometer measured load once per week. **FRT:** Performed the same isometric exercises as the PRT group. Training load was fixed at 70% of the participants maximal strength, as recorded at baseline.
Ris et al [47] (RCT)	2016	Denmark	Participants aged ≥18 years old who have experienced neck pain for ≥6 months and score >10 on the Neck Disability Index. Total sample size at baseline n = 200 (Intervention n = 101 [31.7% male; 68.3% female]; Control n = 99 [19.2% male; 80.8% female]). Sample size at follow-up n = 164 (Intervention n = 89; Control n = 75). Total analysed due to intention-to-treat analysis n = 200. *NB Follow-up gender percentage was not reported in this study*. **Age**: [Mean years (95% CI)]: Exercise Group: 45.1 (42.8; 47.3) Control Group: 45.2(42.9; 47.5) **Cause**: [Traumatic/non-traumatic n (%)]: Exercise Group: 60/41 (59.4%/40.6%) Control Group: 60/39 (60.6%/39.4%) **Duration of Symptoms**: [Mean months (95% CI)]: Exercise Group: 107.3 (87.4; 127.1) Control Group: 108.3 (87.4; 129.2) **Education Level** **Unskilled or no education:** [n (%)]: Exercise Group: 5 (5.0%) Control Group: 11 (11.1%) **Skilled**: [n (%)]: Exercise Group: 84 (83.2%) Control Group: 79 (79.8%) **Academic**: [n (%)]: Exercise Group: 12 (11.9%) Control Group: 9 (9.1%) **Working Situation** **Working full-time**: [n (%)]: Exercise Group: 33 (32.7%) Control Group: 30 (30.3%) **Working part-time**: [n (%)]: Exercise Group: 25 (24.8%) Control Group: 25 (25.2%) **Early retirement**: [n (%)]: Exercise Group: 9 (8.9%) Control Group: 6 (6.1%) **Unemployed**: [n (%)]: Exercise Group: 4 (4.0%) Control Group: 12 (12.1%) **Retired**: [n (%)]: Exercise Group: 6 (5.9%) Control Group: 12 (12.1%) **Sick leave**: [n (%)]: Exercise Group: 17 (16.8%) Control Group: 8 (8.1%) **Under education**: [n (%)]: Exercise Group: 7 (6.9%) Control Group: 6 (6.1%) **Sleep Disturbances** **Sleeping undisturbed**: [n (%)]: Exercise Group: 34 (33.7%) Control Group: 32 (32.3%) **Disturbed ≤ 3 x per night**: [n (%)]: Exercise Group: 44 (43.6%) Control Group: 51 (51.5%) **Disturbed > 3 x per night**: [n (%)]: Exercise Group: 23 (22.7%) Control Group: 16 (16.1%)	PRT combined with graded physical training. Exercises performed twice daily and physical training 3 times weekly for 4 months.	4 sessions of advice and education.	Measures assessed at baseline and at 4-month follow-up.	PPT	Exercise group demonstrated statistically significant improvement in comparison to control at 4-month follow-up bilaterally for cervical spine PPT assessment (left cervical spine *p =* 0.01; right cervical spine *p =* 0.04).	**Exercise Group**: Progressive exercises were individually designed to focus upon: neck flexor and extensor function, standing balance, oculomotor training and neuromuscular function of the shoulder girdle. Graded physical training was created from a self-chosen programme (e.g. walking or cycling), with the initial training level set to 20% below the participant’s capability. This was increased by 20% every second week. Training was aimed at an RPE of 11–14 on a 6–20 Borg Scale. Patients performed exercises twice daily and physical training 3 times weekly for 4 months.

Key

QST = quantitative sensory testing, RCT = randomised controlled trial, TE = therapeutic exercise, MT = manual therapy, PPT = pain pressure threshold, PRT = progressive resistance training

Only one study [47] reported a difference between groups regarding baseline demographic data, which related to a greater percentage (80.8%) of female participants present in the control group.

Regarding population characteristics, all studies reported participant age and gender. Two studies [45, 46] reported height, weight, and BMI data. Two studies [46, 47] reported duration of symptoms and employment data. One study [46] reported job experience and computer usage. One study [47] reported educational level and sleep quality data. Data pertaining to population characteristics are presented in Table 1.

### Population

Excluding the second intervention group (manual therapy) from one study [45] as this was not an outcome of interest, a total of 355 participants enrolled at recruitment across all studies. Due to dropout across all three studies, the total number of participants analysed was 352, as intention-to-treat analysis was utilised in two studies [46, 47]. It was not possible to calculate gender distribution at baseline recruitment, as participants in one study dropped out prior to demographic data collection [45]. However, of the 352 participants included in analysis across all studies, 17.3% were male and 82.7% were female, with a higher proportion of females notably present across all studies, especially in the study by Li et al [46], which was restricted to female participants only.

Eligibility criteria varied across all studies. Minimum duration of neck pain required for participant inclusion varied from ≥12 weeks to >1 year. Two studies required neck pain to be confirmed by official medical diagnosis [46, 47], one study required minimum level of disability on the Neck Disability Index of 10 [47] and one a minimum self-reported pain intensity of 2/10 on a numerical scale of 0–10 [46].

Two studies [45, 46] reported age as mean years (+/- SD). The mean age for participants per group in the study by Bernal-Utrera et al [45] was: control = 36.9 (SD± 2.9) years; TE = 36.8 (SD± 2.9) years. For participants in the study by Li et al: [46] control = 34.1 (SD± 8.2) years; PRT group = 35.6 (SD± 7.9) years; FRT group = 33.7 (SD± 9.0) years. One study [47] reported age data as mean years (95% CI): control = 45.2 (42.9; 47.5) years; TE = 45.1 (42.8; 47.3).

### Intervention

All three studies included active exercise of the neck in the intervention group for their respective trials [45–47]. Reporting of interventions was inconsistent across studies, with one study providing limited detail of intervention protocol [47]. Although the frequency of exercise was consistently reported across the reviewed studies, only one study provided details on the number of sessions provided. A summary of interventions is reported in S4 File.

The duration of intervention ranged from 3 weeks to 4 months, with a frequency of training ranging from twice per day to ≥3 times per week. Exercise intensity was inconsistently reported across the included studies. Two studies included a PRT protocol, one progressed by exercise type [45] and one progressed by an increase in resistance percentage [46], one a PRT protocol combined with graded physical training [47] and one an FRT protocol [46]. One study performed the intervention at the location of participant recruitment [47], with two studies completing the intervention in a home-based environment [45, 46].

Supervision of participants when completing intervention protocols varied across studies, with one providing no supervision [46], one providing 8 x 30-minute sessions of instruction interspersed with the intervention protocol [47] and one providing three sessions (one per week) of individual physiotherapy to ensure correct technique and teach progressions of exercise [45]. Assessors remained blinded in the two studies that included supervision [45, 47], with supervision provided by separate physiotherapists, which mitigated the risk of concealment bias.

Various equipment was utilised across studies, such as Theraband and a handheld dynamometer [46]. Two of the three studies [46, 47] additionally provided advice and education to participants in the intervention group.

Adherence was monitored in two studies [46, 47] and was reported as good in both. One study [45] included a second intervention group that received MT. However, this is not an outcome of interest for this review and, therefore, is not included in the synthesis of results.

### Comparator

Control groups were allocated for all three studies [45–47]. As per the eligibility criteria for this review, no study provided active intervention for comparator groups. One study provided sham manual therapy [45] and two provided advice and education [46, 47].

### Outcome measures

All studies utilised PPTs as the chosen QST modality for the assessment of pain sensitivity. Three assessments are deemed necessary to obtain an accurate estimate of pain sensitivity [48]. One study [45] obtained PPT measurements at the level of the C2 vertebrae spinous process of the cervical spine, calculating a mean value from three measurements for statistical analysis. One study [46] obtained PPT measurements at the painful region of the cervical spine, again calculating a mean value from three measures for statistical analysis. The final study [47] assessed PPT bilaterally at the level of C5/6 vertebrae but did not provide information on the number of measures assessed for statistical analysis.

The length of follow-up varied across studies, with assessment completed at 2 weeks [45, 46], 4 weeks [45, 46], 6 weeks [46], 12 weeks/3 months [45, 46] and 4-months [47].

Although not the primary focus of this study, secondary outcomes (Visual Analogue Scale, Neck Disability Index and Pain Bothersomeness Scale) that may be of interest are briefly reported. These data are presented in S5 File but are not included in the synthesis of results.

### Risk of bias

**Fig 2:**
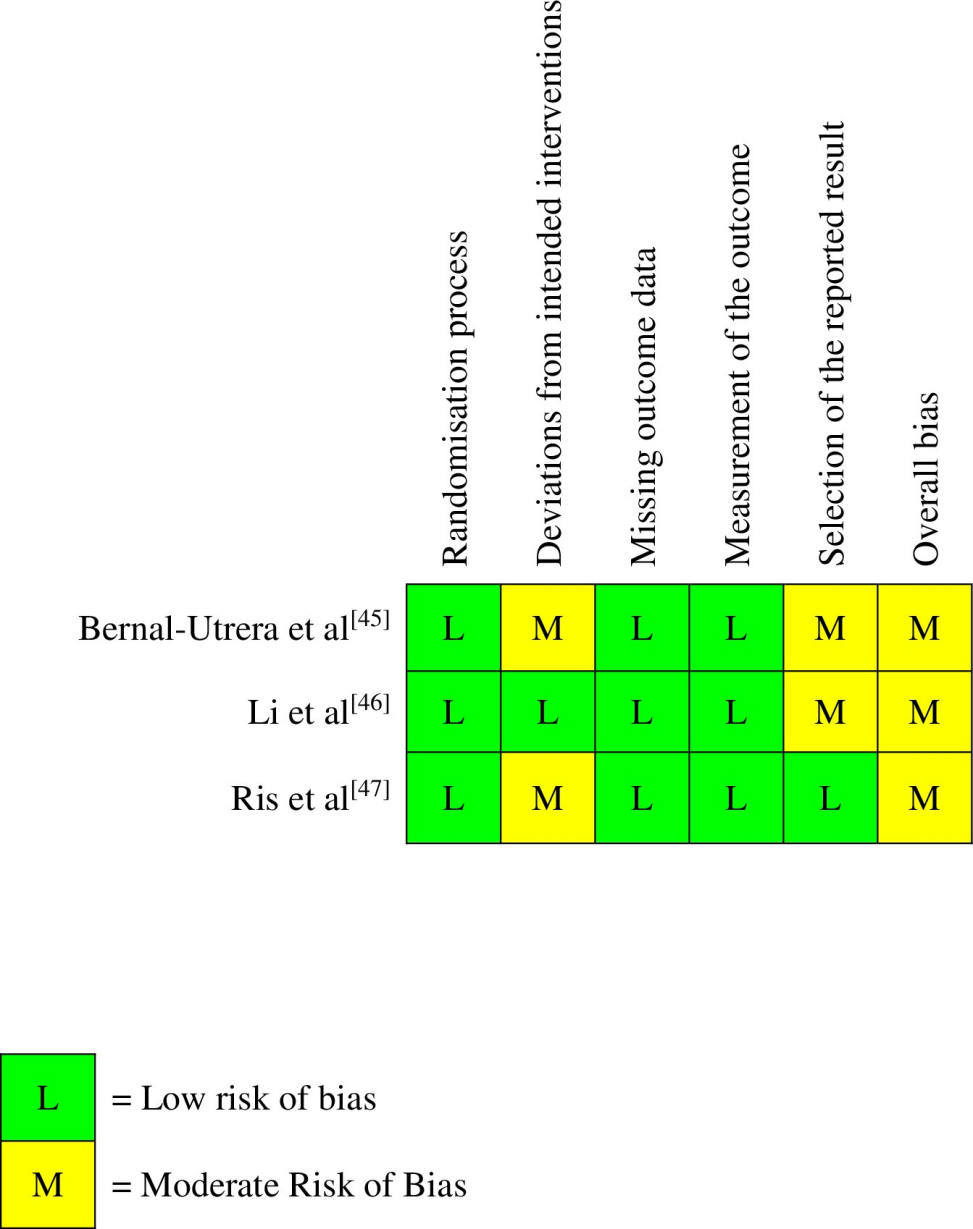
Risk of bias assessment. **Key:** Classification of risk of bias for each included study, as determined by the Cochrane RoB-2 tool. Risk of bias is separated into five different domains that are rated separately, with an overall judgement of risk of bias also provided. All studies were rated as having either low risk of bias, or moderate risk of bias in all domains, with no study rated at a high risk of bias in any domain.

Agreement was achieved between both primary reviewers (DO/FJ) across all domains of the Cochrane RoB-2 tool [31] for all studies. An overall summary of risk of bias for each study is presented in Fig 2.

All three studies were rated at an overall moderate risk of bias with concerns stemming from different domains. The two domains that demonstrated the highest risk of bias across all studies were ‘deviations from intended interventions’ and ‘selection of the reported result’.

Two studies [45, 47] were rated at a moderate risk of bias regarding deviations from intended interventions; one study [45] due to possible knowledge of allocation for participants and failure to assess participant adherence or implement processes to account for this; the other study [47] due to potential awareness of allocation for participants in both groups with subsequent potential deviations from the intended intervention due to significant dropout in the control group. Furthermore, two studies [45, 46] were rated at a moderate risk of bias regarding selection of the reported result due to failure to report a pre-defined study protocol and analysis plan. Although one study [46] reported a registration number for a trial protocol, this protocol number was linked to a different study by different authors, and we were unable to identify a pre-defined protocol for this study. The other study [45] did not report a protocol.

### Certainty of evidence

Based on GRADE recommendations [36], all groupings were initially rated as high-quality evidence due to their respective studies utilising randomised-controlled trial designs, as this is recognised as the gold standard approach [49]. All groupings were downgraded due to risk of bias. As with the RoB-2 analysis, concerns stemmed from possible knowledge of assigned intervention or poor blinding of participants in two studies [45, 47], leading to possible performance bias. Furthermore, high participant dropout potentially consequential to this may have resulted in attrition bias in one study [47]. There were also concerns regarding unidentifiable trial protocol in two studies [45, 46] and the potential introduction of reporting bias because of this.

The total participant attrition rate reported across all studies was 47. One study [45] reported an attrition rate of 4 participants at follow up, one study [46] reported an attrition rate of 7 participants at follow up and one study [47] reported an attrition rate of 36 participants at follow up.

Two groupings were further downgraded due to indirectness resulting from failure to report participant adherence to intervention in one study [45] and stringent eligibility criteria potentially reducing external validity and generalisability of results to the general population in another [46]. Whilst, one study [45] demonstrated a large effect size associated with outcomes of interest for this review, the grouping could not be upgraded as the other study reported no such data. Therefore, one grouping (PRT Combined with Graded Physical Training) was assigned an overall rating of ‘moderate’ quality evidence, and two groupings (PRT and FRT) were ascribed an overall ‘low’ quality of evidence. The overall GRADE rating of each grouping is presented in Table 2.

**Table 2:** GRADE rating for certainty of evidence per study.

Certainty assessment								Number of patients		Effect	Certainty
Grouping	Studies Included	Study design	Risk of bias	Inconsistency	Indirectness	Imprecision	Other considerations	Intervention	Control		
**PRT**	Bernal-Utrera et al [45] Li et al [46]	RCT	Serious	Not serious	Serious	Not serious	Strong association in only one study	61	56	Effect size at follow-up reported in one study: R^2^ = 0.328 *P-*values at follow-up reported in one study: *p*<0.001	⨁⨁◯◯ Low
**FRT**	Li et al [46]	RCT	Serious	Not serious	Serious	Not serious	None	35	36	*P-*values at follow-up: *p*<0.001	⨁⨁◯◯ Low
**PRT Combined with Graded Physical Training**	Ris et al [47]	RCT	Serious	Not serious	Not serious	Not serious	None	101	99	*P-*values at follow-up: *p*<0.005	⨁⨁⨁◯ Moderate

Key

RCT = randomised controlled trial, PRT = progressive resistance training, FRT = fixed resistance training

Participants included in the intervention group of the study by Bernal-Utrera et al [45] exclude participants from the MT group as this is not of interest to this review. Number of patient figures across all studies are based on participants at assessment.

### Publication bias

Due to the low number of studies included within this review, it was not possible to perform statistical analysis for publication bias. Because of this, publication bias could not be assumed and is, therefore, rated as undetected for all studies. It is, therefore, acknowledged that there is a possibility of existence of publication bias and subsequent potential for data within this review to result in reduced precision of conclusions drawn.

### Direction of effect

The direction of effect for all groupings and studies is presented in Table 3. Data in the direction of effect table is calculated based upon intermediate-term follow-up assessment as this was consistently assessed across all studies. The direction of effect assessment demonstrates a positive direction of effect for all studies and groupings. A sign test was not conducted due to the small number of studies included in this review, which would have resulted in an underpowered test [43].

**Table 3:** Direction of effect plot with RoB-2 and GRADE rating.

Intervention	Studies Included	Direction of effect	GRADE
PRT	Bernal-Utrera et al [45] Li et al [46]	▲Small	⨁⨁◯◯
PRT Combined with Graded Physical Training	Ris et al [47]	▲ Medium	⨁⨁⨁◯
FRT	Li et al [46]	▲ Small	⨁⨁◯◯

Key

PRT = progressive resistance training, FRT = fixed resistance training

**Study design:** Randomised controlled trial for all studies.

**Effect direction:** Upward arrow (▲) denotes a positive health impact; downward arrow (▼) denotes a negative health impact; sideways arrows (◂▸) denote no change or conflicting findings.

**Sample size:** Intervention group sample size influences the size of the effect direction arrow. A large arrow (▲) denotes >300 participants; a medium arrow (▲) denotes 50–300 participants; a small arrow (▲) denotes <50 participants. The direction (positive, negative or no difference/conflicting findings) was identified from within written discussion text in each study.

**Risk of Bias:** Rating based upon RoB-2 assessment. Yellow highlight denotes moderate risk of bias.

**GRADE:** GRADE quality of evidence rating per study. ⨁⨁⨁⨁ = high; ⨁⨁⨁ = moderate; ⨁⨁ = low; ⨁ = very low.

### Results of individual studies

The results of the individual studies could not be combined because of wide variation between study and reported effect measures. One study [45] reported R^2^ values to demonstrate the size of effect; the other two [46, 47] did not. All studies [45–47] reported *p*-values to determine statistical significance between groups at follow-up (three and four months). Only one study [45] reported effect sizes for between-groups differences at any stage.

High clinical heterogeneity, primarily regarding duration of neck pain for inclusion, participant gender, study setting, intervention type and duration of intervention, was present across the included studies. Variations in participant demographics, such as gender distribution or age range, as well as clinical characteristics such as the duration of their pain, may impact on the response to treatment. Furthermore, variations in intervention protocols, including different exercise types or dosages, can introduce significant heterogeneity. These differences can lead to disparate outcomes and make it challenging to compare or combine results. Because of this, meta-analysis was not possible. Therefore, a narrative synthesis of results is reported. Although results were planned to be grouped according to outcome measure, as per the published protocol, all studies utilised the same outcome measure for QST (PPTs). Therefore, this method of grouping may have limited the analysis and would have resulted in only a single grouping. Thus, results were instead grouped by exercise type.

### Progressive resistance training

Progressive resistance training was investigated in two trials [45, 46] (117 participants between intervention and control groups at analysis). Progressive resistance training entailed any resistance training programme that was systematically progressed in difficulty by frequency, intensity, duration or type of exercise. One study [45] implemented PRT through increasing exercise load and type on a weekly basis. One study [46] implemented PRT by progressing a percentage of one repetition maximum every two weeks. Across the two trials [45, 46], PRT showed a moderate association. Both studies [45, 46] reported statistically significant improvements (*p* = 0.001 and *p*<0.001 respectively) demonstrating PRT was effective at reducing pain sensitivity in comparison to control at intermediate-term follow-up (12-weeks/3-months respectively), with one study [45] also reporting a large effect size in support of this (R^2^ = 0.328), where R^2^>0.14 is considered as a large effect size. The other study did not report the effect size. Both studies indicated a positive direction of effect at intermediate-term follow-up.

One study [46] reported statistically significant short-term improvements in comparison to control at weeks 2 (*p* = 0.017), 4 (*p*<0.001) and 6 (*p*<0.001), whereas the other study [45] did not (week 2: *p =* 0.991; week 4: *p* = 0.415), although a medium effect size (*R*^*2*^ = 0.082) was noted at week 4 assessment for increased PPTs by this study [45]. One study [45] also reported a significant intragroup difference for PRT at week 12 (*p* = 0.001), but not at weeks 2 (*p* = 0.729) or 4 (*p* = 0.061). Both studies demonstrated an overall moderate risk of bias, with issues primarily stemming from concerns regarding deviations from intended intervention and possible performance bias [45] and risk of bias in selection of the reported result [45, 46]. This exercise grouping demonstrated low-quality evidence based on GRADE assessment due to risk of bias issues in both studies, and indirectness issues, including poor generalisability [46] and lack of reporting regarding adherence to the intervention [45].

Based on low to moderate quality of evidence (GRADE), PRT is effective at increasing PPTs at intermediate-term follow-up.

### Progressive resistance training combined with graded physical training

Progressive resistance training combined with graded physical training was investigated in one study [47] (200 participants between intervention and control groups at analysis), which demonstrated a statistically significant improvement (left cervical spine: *p* = 0.001; right cervical spine: *p* = 0.004) in comparison to control at 4-month follow-up for PPTs. A positive direction of effect was indicated. No effect size was reported. The study demonstrated a moderate risk of bias due to some concerns regarding performance and attrition bias. This grouping was rated as moderate quality of evidence based on GRADE assessment due to concerns regarding risk of bias.

Based on moderate-quality evidence (GRADE), PRT combined with graded physical training is effective at increasing PPTs at intermediate-term follow-up.

### Fixed resistance training

Fixed resistance training was investigated in one study [46] (71 participants between intervention and control groups at analysis). The findings demonstrated a statistically significant improvement for FRT in comparison to control at weeks 4 (*p* = 0.001), week 6 (*p*<0.001) and at 3-month follow-up (*p*<0.001), but not at week 2 (*p* = 0.075). A positive direction of effect was indicated at intermediate-term follow-up. No effect size was reported. The study was rated at an overall moderate risk of bias due to concerns regarding selection of the reported result. This grouping was rated as low quality of evidence based on GRADE assessment due to concerns regarding risk of bias and potentially poor generalisability of results.

Based on low-quality evidence (GRADE), FRT is effective for increasing PPTs at short-term (from week 4) and intermediate-term follow-up.

## Discussion

This review was conducted to identify the effects of neck-specific exercise when performed for ≥2 weeks in adults with chronic neck pain as measured by QST, which to our knowledge, is the first to investigate this. Four exercise protocols from three studies were evaluated. Population, eligibility criteria and outcomes were generally well described across studies. However, intervention protocols were inconsistently described across studies which limits an in-depth understanding of such interventions. Furthermore, high clinical heterogeneity was present across the included studies, preventing pooling of data and overall estimation of effect through meta-analysis. Therefore, data were narratively synthesised and grouped by exercise type. The results demonstrate different exercise types to be beneficial for the reduction of pressure pain sensitivity at different time-points.

A positive direction of effect and statistically significant increases in PPTs were demonstrated for all exercise interventions at intermediate-term follow-up, regardless of grouping, demonstrating that exercise is an effective modality for the reduction of pressure pain sensitivity for individuals with chronic neck pain. No intervention demonstrated a negative effect on pain sensitivity. PRT was rated with low level of evidence, PRT combined with graded physical training with moderate quality of evidence and FRT with low quality of evidence. A large effect size was reported by one study in support of PRT, which demonstrates strong benefit for this exercise type. However, as it was not possible to calculate estimates of effect for all groups or to assess dosage, comparison could not be made regarding size of improvement and which training modality may be most effective at changing pressure pain sensitivity in the intermediate term.

Evidence was equivocal regarding the short-term effects of exercise. Findings conflicted for PRT, as one study demonstrated short-term improvements [46], but the other [45] did not (though a medium effect size was reported at 4-week assessment, suggesting some small improvements were being demonstrated at this time-point), meaning that PRT cannot be recommended for short-term improvements due to such disagreement between studies. Some low-quality evidence demonstrated benefit for the use of FRT for short-term (from week 4) improvement, though only one study was included in this exercise grouping, meaning there is no additional data to support or refute this, as with PRT findings. Further, data demonstrating short-term improvements across both PRT and FRT groups were from the same primary study [46], which utilised the same exercise type and dosage, with progression achieved through increasing percentage resistance for the PRT group.

Several explanations for short-term improvements may be plausible. This may be due to: firstly, the exercise type and dosage included within the study that noted improvement [46]; secondly, variations in sample characteristics and inclusion criteria between studies, with the study demonstrating benefit [46] only including female participants with a longer pain duration and chronic neck pain related to computer use and posture; thirdly, the study demonstrating short-term benefit [46] provided additional advice and education to participants, whereas the other did not. However, this may be considered less likely as advice and education only is not deemed beneficial for neck pain [50, 51], unless used for people with whiplash associated disorder (WAD) [52]. The study that found benefit excluded WAD patients.

Overall, the results provide low to moderate quality evidence that all types of neck-specific exercise included in this review may be beneficial for the reduction of cervical pressure pain sensitivity at the intermediate-term, but it is unclear which modality is most effective. Based on the quality of evidence, PRT combined with graded physical training demonstrated an improved certainty of findings, though only one study was included in this grouping. Results were equivocal regarding short-term effects for PRT, with some low-quality evidence suggesting that FRT may be beneficial for short-term (from week 4) improvement in cervical pain sensitivity. Therefore, clinicians should not base their selection of exercise type purely on findings of this review given the current uncertainty. They may look to consider additional factors such as patient ability to perform specific exercises, the type of neuromuscular adaptations likely to be induced by different exercises, activity level/exercise experience and pain levels, in addition to other relevant demographic data, to also assist in guiding treatment decisions.

Our findings are consistent with a review by Bertozzi et al [16] that considered the effects of exercise on chronic neck pain, as assessed by non-QST measures, concluding that exercise is effective in the intermediate-term. The same review also concluded that exercise is beneficial for short-term improvement, for which our findings are not as definitive, but indicate the use of FRT. Another study by Wilhelm et al [15], which considered the effects of exercise on neck pain in acute, sub-acute and chronic populations with non-QST parameters, also supports the findings of this review, similarly concluding that exercise is effective at intermediate to long-term follow-up. However, it is of note that these studies did not utilise QST outcomes and participants were of varying pain chronicity.

In contrast to our review, Price et al [26] demonstrated no effect for exercise on chronic neck pain at the intermediate-term assessment. However, again, this study did not utilise QST as an outcome for the assessment of pain and numerous primary studies in the study by Price et al [26] included an active comparator, which may have influenced findings and was a reason for the exclusion of active comparators in our review.

For comparison with previous research utilising QST, the findings of this review fall in line with research by Lyng et al [18] investigating the effects of exercise on shoulder pain, which concluded that exercise beneficially modulated PPTs.

### Risk of bias and certainty of evidence

The overall risk of bias for all three included studies was rated at moderate. Common issues related to poor reporting of pre-defined study protocols, participant attrition rates and blinding processes, whereby participants were likely aware of their assigned intervention. Although this may have been caused by difficulty disguising interventions due to their nature (e.g., exercise vs inactive control), there are concerns regarding the potential impacts on the internal validity of the studies, especially when potential knowledge of intervention was not accounted for, and the subsequent findings of the effects of exercise; something that has also been noted in prior reviews [18].

Further to risk of bias assessment, the certainty of evidence was rated at either low or moderate for all groupings. Shortcomings related to the aforementioned risks of bias and indirectness. Only one study reported an effect size for the primary outcome. Reporting of this, or presentation of data pertaining to calculation of the effect size may have been beneficial for the two studies and may have resulted in an improved certainty of evidence rating within the exercise groupings which would have assisted the synthesis of our findings.

### Strengths and limitations

This systematic review has various strengths. Firstly, the study and intended methods were prospectively registered as a publicly accessible protocol on the PROSPERO database. The reporting of this study also followed both PRISMA and SWiM reporting guidelines to improve reporting quality and transparency. Although some deviations from protocol occurred, these were transparently reported. Data selection was independently performed by two reviewers, whilst data extraction, risk of bias and certainty of evidence assessment were performed by one reviewer and then independently ratified by a second reviewer, with discussion between the reviewers if required.

Limitations were present within this review. Due to a high degree of clinical heterogeneity (e.g., duration of neck pain for inclusion, participant gender, study setting, intervention type and duration of intervention) and differing effect measures across studies, meta-analysis and estimation of effect, which would have been a superior method for combining results [53], was not possible, consequently limiting the strength of answer to the review question. Additionally, although a thorough literature search was conducted, the stringent eligibility criteria, including restriction to articles published in the English language, may have caused exclusion of appropriate articles. Only three studies were included in the review, resulting in conclusions being drawn from a limited sample of data. It was also not possible to assess publication bias, which may have influenced conclusions [54].

Regarding the dataset from which conclusions of this review are drawn, additional limitations are present. It was not possible to assess long-term effects as none of the included studies investigated this timeframe for follow-up. The small sample sizes across the included studies, combined with strict eligibility criteria of some studies, somewhat limits the external validity and generalisability of findings to the wider population. Gender distribution favoured females across the three studies, again potentially limiting generalisability. Furthermore, the average age of participants only fell within the age range in which CNSNP is most prevalent [2] in one study. Thus, the results may not directly relate to the greatest proportion of the population for which CNSNP is prevalent. Considering such limitations, it is difficult to generalise the results of this study to a single population and to understand whether TE is beneficial for long-term improvement.

Furthermore, limitation is present when comparing this review to previous systematic reviews due to heterogeneity between populations and outcome measures. Therefore, comparison with previous research should be considered with caution. Our systematic review also has limitations that may affect the generalisability of the results. Firstly, the sample sizes varied across the included studies, with some having smaller cohorts, potentially impacting the robustness of the findings. Future research with larger, more representative samples is needed to enhance the reliability and generalizability of our findings. Secondly, the gender distribution within the study populations was not consistently reported or balanced across all studies, raising concerns about the extrapolation of findings to populations with different gender compositions. Future studies should ensure adequate representation of both males and females to enhance the applicability of the results. Lastly, the age range of participants included in the studies varied, potentially impacting the generalisability of the findings to populations with distinct age demographics. Future research should aim to elucidate how findings extrapolate to specific age cohorts to better inform clinical practice across the lifespan.

From a physiotherapist’s perspective, exercise adjustments such as modifying resistance, duration, and intensity can customise these exercises to suit individual needs, abilities, and conditions. Additionally, a thorough assessment and personalised exercise plan that takes into account factors such as age, fitness level, and any other health issues ensures that the exercises are applied safely and effectively across various populations, promoting therapeutic benefits while minimising the risk of injury or worsening of symptoms.

### Implications

Exercise is currently commonly utilised in standardised care for chronic neck pain as part of a multi-modal management approach [6]. The current findings build on previous evidence [15, 16] and support its use. Our results do not provide clear conclusions as to the most effective exercise type, especially for intermediate-term improvements, which does not help to develop evidence of current clinical guidelines [6]. Moreover, certain protocols utilised equipment that may not be typically available to care providers or patients (e.g. dynamometers) and may require baseline standards of fitness (e.g. for graded physical training). Thus, it may be prudent for clinicians to select exercise type based on patient requirements, capability and equipment availability until firm conclusions can be drawn regarding size of effect. Consideration of the natural covariate factors that may limit generalisability, such as participant gender and age, should also be considered by practitioners when using findings of this review to inform practice.

The use of QST in this study provides an initial understanding of pain response in relation to exercise measured using a more objective method. Furthermore, if more robust future research can develop current understanding of the benefits of exercise, the utilisation of QST may provide a more in depth understanding of pain mechanisms that are not identifiable alone using patient reported measures, which may allow for understanding of subgroups of patient who may benefit from certain types of exercise over another. This may then implicate whether the use of QST in clinical practice would be beneficial for greater individualisation of pain management and treatment selection.

Based upon these clinical implications, future research should include larger samples with a balanced gender distribution. Long-term follow-up would also help provide an understanding of the long-term effects of neck-specific exercise and further inform clinical practice. Additionally, comprehensive data should be openly reported to enable calculation of more meaningful statistics, such as size of effect, allowing future reviews to develop an understanding of which exercise modality demonstrates the most benefit.

## Conclusion

Definitive evidence is currently lacking with regard to whether exercise is beneficial for the reduction of pressure pain sensitivity in adults with chronic neck pain. There may be some indications that certain types of exercise could be beneficial at certain timepoints. However, further research is required to corroborate these findings.

Based on low-quality evidence and supported by one study, FRT may be effective for short-term (from week 4) improvement. At intermediate-term follow-up all exercise groupings demonstrated improvement based on low to moderate quality evidence (supported by three studies). PRT combined with graded physical training demonstrated the highest quality of evidence of the exercise groupings at intermediate-term follow-up (based on one study). Due to the inability to complete meta-analysis and calculate overall effect estimates, it was not possible to infer which exercise modality is most beneficial. Furthermore, due to methodological limitations within the primary research within this review, robust conclusions cannot be drawn. Future research should aim to conduct studies with greater sample size, improved gender distribution and consider long-term effects to gain a greater understanding of the effects of exercise on chronic neck pain as measured by QST.

## Supporting information

S1 File Search strategy. (DOCX)

S2 File Data extraction forms. (XLSX)

S3 File Risk of bias assessment. (XLSX)

S4 File Summary of interventions. (DOCX)

S5 File PRISMA 2020 checklist. (DOCX)

S6 File SWiM checklist. (PDF)

## References

[1] Miyamoto GC, Lin C-WC, Cabral CMN, et al. Cost-effectiveness of exercise therapy in the treatment of non-specific neck pain and low back pain: a systematic review with meta-analysis. *Br J Sports Med*. 2019;53:172–181. doi: 10.1136/bjsports-2017-098765 29678893

[2] Safiri S, Kolahi AA, Hoy D, et al. Global, regional, and national burden of neck pain in the general population, 1990–2017: systematic analysis of the Global Burden of Disease Study 2017. *BMJ*. 2020;368:1–11. doi: 10.1136/bmj.m791 32217608 PMC7249252

[3] Hoy DG, Protani M, De R, et al. The epidemiology of neck pain. *Best Practice & Research Clinical Rheumatology*. 2010;24:783–792. doi: 10.1016/j.berh.2011.01.019 21665126

[4] Parikh P, Santaguida P, Macdermid J, et al. Comparison of CPG’s for the diagnosis, prognosis and management of non-specific neck pain: a systematic review. *BMC Musculoskeletal Disorders*. 2019;20:1–13. 30764789 10.1186/s12891-019-2441-3 PMC6376764

[5] Coulter ID, Crawford C, Vernon H, et al. Manipulation and Mobilization for Treating Chronic Nonspecific Neck Pain: A Systematic Review and Meta-Analysis for an Appropriateness Panel. *Pain Physician*. 2019;22:E55–E70. 30921975 PMC6800035

[6] National Institute of Health and Care Excellence (NICE). Neck pain–non-specific. 2022. Available at: https://www.nice.org.uk/guidance/qs86. Accessed July 1, 2022.

[7] Hidalgo B, Hall T, Bossert J, et al. The efficacy of manual therapy and exercise for treating non-specific neck pain: A systematic review. *Journal of Back and Musculoskeletal Rehabilitation*. 2017;30:1149–1169. doi: 10.3233/BMR-169615 28826164 PMC5814665

[8] Binder A. The diagnosis and treatment of nonspecific neck pain and whiplash. *Eura Medicophys*. 2007;43:79–89. 17369782

[9] McLean SM, May S, Klaber-Moffett J, et al. Risk factors for the onset of non-specific neck pain: a systematic review. *J Epidemiol Community Health*. 2010;64:565–572. doi: 10.1136/jech.2009.090720 20466711

[10] Cohen SP. Epidemiology, Diagnosis, and Treatment of Neck Pain. *Mayo Clinic Proceedings*. 2015;90:284–299. doi: 10.1016/j.mayocp.2014.09.008 25659245

[11] Jahre H, Grotle M, Smedbråten K, et al. Risk factors for non-specific neck pain in young adults. A systematic review. *BMC Musculoskelet Disord*. 2020;21:1–12. doi: 10.1186/s12891-020-03379-y 32517732 PMC7285427

[12] Kraemer WJ, Ratamess NA, French DN. Resistance training for health and performance. *Curr Sports Med Rep*. 2002;1:165–171. doi: 10.1249/00149619-200206000-00007 12831709

[13] Louw S, Makwela S, Manas L, et al. Effectiveness of exercise in office workers with neck pain: A systematic review and meta-analysis. *S Afr J Physiother*. 2017;73:1–11. doi: 10.4102/sajp.v73i1.392 30135909 PMC6093121

[14] Sihawong R, Janwantanakul P, Sitthipornvorakul E, et al. Exercise Therapy for Office Workers With Nonspecific Neck Pain: A Systematic Review. *Journal of Manipulative and Physiological Therapeutics*. 2011;34:62–71. doi: 10.1016/j.jmpt.2010.11.005 21237409

[15] Wilhelm MP, Donaldson M, Griswold D, et al. The Effects of Exercise Dosage on Neck-Related Pain and Disability: A Systematic Review With Meta-analysis. *Journal of Orthopaedic & Sports Physical Therapy*. 2020; 50:607–621. doi: 10.2519/jospt.2020.9155 33131392

[16] Bertozzi L, Gardenghi I, Turoni F, et al. Effect of Therapeutic Exercise on Pain and Disability in the Management of Chronic Nonspecific Neck Pain: Systematic Review and Meta-Analysis of Randomized Trials. *Physical Therapy*. 2013;93:1026–1036. doi: 10.2522/ptj.20120412 23559524

[17] Backonja M, Attal N, Baron R, et al. Value of quantitative sensory testing in neurological and pain disorders: NeuPSIG consensus. *PAIN*. 2013;154:1807–1819. doi: 10.1016/j.pain.2013.05.047 23742795

[18] Lyng KD, Thorsen JBB, Larsen DB, et al. The Modulatory Effect of Quantitative Sensory Testing in Shoulder Pain: A Systematic Review and Meta-Analysis. *Pain Med*. 2022;23:733–744. doi: 10.1093/pm/pnab155 33905508

[19] Marcuzzi A, Wrigley PJ, Dean CM, et al. The long-term reliability of static and dynamic quantitative sensory testing in healthy individuals. *PAIN*. 2017;158:1217–1223. doi: 10.1097/j.pain.0000000000000901 28328574

[20] Mackey IG, Dixon EA, Johnson K, et al. Dynamic Quantitative Sensory Testing to Characterize Central Pain Processing. *J Vis Exp*. 2017;120:1–9. doi: 10.3791/54452 28287532 PMC5407598

[21] La Touche R, Martínez García S, Serrano García B, et al. Effect of Manual Therapy and Therapeutic Exercise Applied to the Cervical Region on Pain and Pressure Pain Sensitivity in Patients with Temporomandibular Disorders: A Systematic Review and Meta-analysis. *Pain Med*. 2020;21:2373–2384. doi: 10.1093/pm/pnaa021 32181811

[22] Moher D, Liberati A, Tetzlaff J, et al. Preferred reporting items for systematic reviews and meta-analyses: the PRISMA statement. *BMJ*. 2009;339: 2535–b2535. 10.1136/bmj.b2535 PMC2714657 19622551

[23] Page MJ, McKenzie JE, Bossuyt PM, et al. The PRISMA 2020 statement: an updated guideline for reporting systematic reviews. *BMJ*. 2021;372:1–9. 10.1136/bmj.n71 PMC8005924 33782057

[24] Huang X, Lin J, Demner-Fushman D. Evaluation of PICO as a Knowledge Representation for Clinical Questions. *AMIA Annu Symp Proc*. 2006:359–363. 17238363 PMC1839740

[25] Methley AM, Campbell S, Chew-Graham C, et al. PICO, PICOS and SPIDER: a comparison study of specificity and sensitivity in three search tools for qualitative systematic reviews. *BMC Health Services Research*. 2014;14:1–10. 25413154 10.1186/s12913-014-0579-0 PMC4310146

[26] Price J, Rushton A, Tyros I, et al. Effectiveness and optimal dosage of exercise training for chronic non-specific neck pain: A systematic review with a narrative synthesis. *PLOS ONE*. 2020;15:1–32. doi: 10.1371/journal.pone.0234511 32520970 PMC7286530

[27] Furlan AD, Malmivaara A, Chou R, et al. 2015 Updated Method Guideline for Systematic Reviews in the Cochrane Back and Neck Group. *Spine*. 2015;40:1660–1673. doi: 10.1097/BRS.0000000000001061 26208232

[28] Tampin B, Slater H, Hall T, et al. Quantitative sensory testing somatosensory profiles in patients with cervical radiculopathy are distinct from those in patients with nonspecific neck-arm pain. *Pain*. 2012;153:2403–2414. doi: 10.1016/j.pain.2012.08.007 22980746

[29] Halperin I, Pyne DB, Martin DT. Threats to Internal Validity in Exercise Science: A Review of Overlooked Confounding Variables. *International Journal of Sports Physiology and Performance*. 2015;10:823–829. doi: 10.1123/ijspp.2014-0566 25756869

[30] Clarivate Analytics. EndNote 20. 2013, ed2020.

[31] Sterne JAC, Savović J, Page MJ, et al. RoB 2: a revised tool for assessing risk of bias in randomised trials. *BMJ*. 2019;366:1–8. doi: 10.1136/bmj.l4898 31462531

[32] Page MJ, McKenzie JE, Higgins JPT. Tools for assessing risk of reporting biases in studies and syntheses of studies: a systematic review. *BMJ Open*. 2018;8:1–16. doi: 10.1136/bmjopen-2017-019703 29540417 PMC5857645

[33] Flemyng E, Dwan K, Moore TH, et al. Risk of Bias 2 in Cochrane Reviews: a phased approach for the introduction of new methodology. *Cochrane Database of Systematic Reviews*. 2020;11: 1–11. doi: 10.1002/14651858.ED000148 33215687 PMC10284096

[34] Minozzi S, Cinquini M, Gianola S, et al. The revised Cochrane risk of bias tool for randomized trials (RoB 2) showed low interrater reliability and challenges in its application. *Journal of Clinical Epidemiology*. 2020;126:37–44. doi: 10.1016/j.jclinepi.2020.06.015 32562833

[35] Guyatt GH, Oxman AD, Vist GE, et al. GRADE: an emerging consensus on rating quality of evidence and strength of recommendations. *BMJ*. 2008;336:924–926. doi: 10.1136/bmj.39489.470347.AD 18436948 PMC2335261

[36] Schünemann H, Brożek J, Guyatt G, et al. GRADE handbook for grading quality of evidence and strength of recommendations. Updated October 2013. The GRADE Working Group, 2013.

[37] GRADEpro GDT: GRADEpro Guideline Development Tool [computer programme]. McMaster University and Evidence Prime; 2022.

[38] Schünemann HJ, Higgins JPT, Vist GE, et al. Chapter 14: Completing ‘Summary of findings’ tables and grading the certainty of the evidence. In: Higgins JPT, Thomas J, Chandler J, et al eds. *Cochrane Handbook for Systematic Reviews of Interventions version 6*.*3* *(*updated February 2022). Chichester (UK): John Wiley & Sons, 2019.

[39] Keshavarz H, Fitzpatrick-Lewis D, Streiner DL, et al. Screening for depression: a systematic review and meta-analysis. *CMAJ Open*. 2013;1:E159–E167. doi: 10.9778/cmajo.20130030 25077118 PMC3986010

[40] Pollock A, Farmer SE, Brady MC, et al. An algorithm was developed to assign GRADE levels of evidence to comparisons within systematic reviews. *Journal of Clinical Epidemiology*. 2016;70:106–110. doi: 10.1016/j.jclinepi.2015.08.013 26341023 PMC4742519

[41] Higgins JPT, Thomas J, Chandler J, et al. eds. *Cochrane Handbook for Systematic Reviews of Interventions version 6*.*3* (updated February 2022). Cochrane, 2022.

[42] Campbell M, McKenzie JE, Sowden A, et al. Synthesis without meta-analysis (SWiM) in systematic reviews: reporting guideline. *BMJ*. 2020;368:1–6. doi: 10.1136/bmj.l6890 31948937 PMC7190266

[43] Boon MH, Thomson H. The effect direction plot revisited: Application of the 2019 Cochrane Handbook guidance on alternative synthesis methods. *Research Synthesis Methods*. 2021;12:29–33. doi: 10.1002/jrsm.1458 32979023 PMC7821279

[44] McKenzie JE, Brennan SE. Chapter 12: Synthesizing and presenting findings using other methods. In: Higgins JPT, Thomas J, Chandler J, et al. eds. *Cochrane Handbook for Systematic Reviews of Interventions version 6*.*3* *(updated February 2022)*. Cochrane, 2022.

[45] Bernal-Utrera C, Gonzalez-Gerez JJ, Anarte-Lazo E, et al. Manual therapy versus therapeutic exercise in non-specific chronic neck pain: a randomized controlled trial. *Trials*. 2020;21:1–10. 32723399 10.1186/s13063-020-04610-w PMC7385865

[46] Li X, Lin C, Liu C, et al. Comparison of the effectiveness of resistance training in women with chronic computer-related neck pain: a randomized controlled study. *Int Arch Occup Environ Health*. 2017;90:673–683. doi: 10.1007/s00420-017-1230-2 28528354

[47] Ris I, Søgaard K, Gram B, et al. Does a combination of physical training, specific exercises and pain education improve health-related quality of life in patients with chronic neck pain? A randomised control trial with a 4-month follow up. *Man Ther*. 2016;26:132–140. doi: 10.1016/j.math.2016.08.004 27598552

[48] Walton DM, Levesque L, Payne M, et al. Clinical Pressure Pain Threshold Testing in Neck Pain: Comparing Protocols, Responsiveness, and Association With Psychological Variables. *Physical Therapy*. 2014;94:827–837. doi: 10.2522/ptj.20130369 24557645 PMC4040424

[49] Schulz KF, Altman DG, Moher D. CONSORT 2010 Statement: updated guidelines for reporting parallel group randomised trials. *BMJ*. 2010;340, c332–c332. doi: 10.1136/bmj.c332 20332509 PMC2844940

[50] Haines T, Gross AR, Burnie S, et al. A Cochrane review of patient education for neck pain. *The Spine Journal*. 2009;9:859–871. doi: 10.1016/j.spinee.2009.04.019 19596214

[51] Gross A, Forget M, George KS, et al. Patient education for neck pain. *Cochrane Database of Systematic Reviews*. 2012;3:1–101. doi: 10.1002/14651858.CD005106.pub4 22419306 PMC12042649

[52] Yu H, Côté P, Southerst D, et al. Does structured patient education improve the recovery and clinical outcomes of patients with neck pain? A systematic review from the Ontario Protocol for Traffic Injury Management (OPTIMa) Collaboration. *The Spine Journal*. 2016;16:1524–1540. doi: 10.1016/j.spinee.2014.03.039 24704678

[53] Fagard RH, Staessen JA, Thijs L. Advantages and disadvantages of the meta-analysis approach. *Journal of Hypertension*. 1996;14:S9–12. doi: 10.1097/00004872-199609002-00004 8934372

[54] Cooper H, Hedges LV, Valentine JC. *The Handbook of Research Synthesis and Meta-Analysis*. Russell Sage Foundation; 2009.

