# Supplementary material for: The effects of neck exercise in comparison to passive or no intervention on quantitative sensory testing measurements in adults with chronic neck pain: A systematic review
Source: PLoS One. 2024 May 3;19(5):e0303166. doi: 10.1371/journal.pone.0303166 (PMC11068209; doi:10.1371/journal.pone.0303166)
Supplement: S1 File — (DOCX) [file pone.0303166.s001.docx]

**Supp file 1: Search Strategies for all Databases, Search Engines and Unpublished Literature Databases**

**AMED & SPORTDiscus (via Ebscohost)**

Exercis* **OR** training **OR** “exercise training” **OR** “motor control” **OR** “physical activit*” **OR** “physical exercise” **OR** resistance **OR** strength **OR** endurance **OR** exercise **OR** stretching **OR** pilates **OR** “Core stab*” **OR** yoga

**AND**

“Neck pain” **OR** “neck injur*” **OR** neckache **OR** “neck trauma” **OR** “cervical pain” **OR** “cervical injur*” **OR “**cervical trauma” **OR** cervicodynia **OR** cervicalgia **OR “**chronic non-specific neck pain” **OR “**chronic neck pain” **OR** “chronic nonspecific neck pain” **OR** “nonspecific neck pain” **OR** whiplash **OR “**whiplash injur*”

**AND**

“Quantitative sensory testing” **OR “**sensory thresholds/” **OR** “pain thresholds/” **OR** threshold* **OR** “detection threshold” **OR** thermal **OR** QST **OR** “pain sensitisation” **OR** “pain sensitization” **OR** “pain modulation” **OR** “pain tolerance” **OR** “temporal summation” **OR** “conditioned pain modulation” **OR** algometer **OR** [allodynia](https://www.sciencedirect.com/topics/medicine-and-dentistry/allodynia) **OR** hyperalgesia

**AND**

“Randomi* controlled trial” **OR** “Non-randomi* controlled trial” **OR** “Control trial” **OR** Intervention **OR** “Clinical trial*” **OR** “comparison group” **OR** “control group” **OR** non-random

**CINAHL**

| # | Query | Limiters/Expanders | Last Run Via |
| --- | --- | --- | --- |
| S1  S2  S3  S4  S5  S6  S7  S8  S9  S10  S11  S12  S13  S14  S15  S16  S17  S18  S19  S20  S21  S22  S23  S24  S25  S26  S27  S28  S29  S30  S31  S32  S33  S34  S35  S36  S37  S38  S39  S40  S41  S42  S43  S44  S45  S46  S47  S48  S49  S50  S51  S52  S53  S54  S55  S56  S57  S58  S59 | "Exercis*"  "training"  "exercise training"  "motor control"  "physical activit*"  "physical exercise"  "resistance"  "strength"  "endurance"  "exercise"  "stretching"  "pilates"  “Core stab*"  "yoga"  S1 OR S2 OR S3 OR S4 OR S5 OR S6 OR S7 OR S8 OR S9 OR S10 OR S11 OR S12 OR S13 OR S14  "Neck pain"  "neck injur*"  "neckache"  “neck trauma"  "cervical pain"  "cervical injur*"  "cervical trauma"  "cervicodynia"  "cervicalgia"  "chronic non-specific neck pain"  "chronic neck pain"  "chronic nonspecific neck pain"  "nonspecific neck pain"  "whiplash"  "whiplash injur*"  S16 OR S17 OR S18 OR S19 OR S20 OR S21 OR S22 OR S23 OR S24 OR S25 OR S26 OR S27 OR S28 OR S29 OR S30  "Quantitative sensory testing"  "sensory thresholds/"  "pain thresholds/"  "threshold*"  "detection threshold”  "thermal"  "QST"  "pain sensitisation"  "pain sensitization"  "pain modulation"  "pain tolerance"  "temporal summation"  "conditioned pain modulation"  "algometer"  "allodynia"  "hyperalgesia"  S32 OR S33 OR S34 OR S35 OR S36 OR S37 OR S38 OR S39 OR S40 OR S41 OR S42 OR S43 OR S44 OR S45 OR S46 OR S47  "Randomi* controlled trial"  "Non-randomi* controlled trial"  "Control trial"  "Intervention"  "Clinical trial*"  "comparison group"  "control group"  "non-random"  S49 OR S50 OR S51 OR S52 OR S53 OR S54 OR S55 OR S56  S15 AND S31 AND S48 AND S57  S15 AND S31 AND S48 AND S57 | Expanders - Apply equivalent subjects Search modes - Boolean/Phrase | Interface - EBSCOhost Research Databases Search Screen - Advanced Search Database - CINAHL Plus |

**Embase (via Ovid)**

Exercis*.mp **OR** training **OR** exercise training.mp **OR** motor control.mp **OR** physical activit*.mp **OR** physical exercise.mp **OR** resistance.mp **OR** strength.mp **OR** endurance.mp **OR** exercise.mp **OR** stretching.mp **OR** pilates.mp **OR** Core stab* **OR** yoga

**AND**

Neck pain.mp **OR** neck injur*.mp **OR** neckache.mp **OR** neck trauma.mp **OR** cervical pain **OR** cervical injur*.mp **OR** cervical trauma **OR** cervicodynia.mp **OR** cervicalgia **OR** chronic non-specific neck pain.mp **OR** chronic neck pain.mp **OR** chronic nonspecific neck pain.mp **OR** nonspecific neck pain.mp **OR** whiplash.mp **OR** whiplash injur*.mp

**AND**

Quantitative sensory testing.mp **OR** sensory thresholds/ **OR** pain thresholds/ **OR** threshold*.mp **OR** detection threshold.mp **OR** thermal.mp **OR** QST.mp **OR** pain sensitisation.mp **OR** pain sensitization.mp **OR** pain modulation.mp **OR** pain tolerance.mp **OR** temporal summation.mp **OR** conditioned pain modulation.mp **OR** algometer.mp **OR** [allodynia](https://www.sciencedirect.com/topics/medicine-and-dentistry/allodynia).mp **OR** [hyperalgesia](https://www.sciencedirect.com/topics/medicine-and-dentistry/hyperpathia).mp

**AND**

Randomi* controlled trial.mp **OR** Non-randomi* controlled trial.mp **OR** Control trial.mp **OR** Intervention.mp **OR** Clinical trial*.mp **OR** comparison group.mp **OR** control group.mp **OR** non-random.mp

**Medline**

Exercis*.mp **OR** training **OR** exercise training.mp **OR** motor control.mp **OR** physical activit*.mp **OR** physical exercise.mp **OR** resistance.mp **OR** strength.mp **OR** endurance.mp **OR** exercise.mp **OR** stretching.mp **OR** pilates.mp **OR** Core stab* **OR** yoga

**AND**

Neck pain.mp **OR** neck injur*.mp **OR** neckache.mp **OR** neck trauma.mp **OR** cervical pain **OR** cervical injur*.mp **OR** cervical trauma **OR** cervicodynia.mp **OR** cervicalgia **OR** chronic non-specific neck pain.mp **OR** chronic neck pain.mp **OR** chronic nonspecific neck pain.mp **OR** nonspecific neck pain.mp **OR** whiplash.mp **OR** whiplash injur*.mp

**AND**

Quantitative sensory testing.mp **OR** sensory thresholds/ **OR** pain thresholds/ **OR** threshold*.mp **OR** detection threshold.mp **OR** thermal.mp **OR** QST.mp **OR** pain sensitisation.mp **OR** pain sensitization.mp **OR** pain modulation.mp **OR** pain tolerance.mp **OR** temporal summation.mp **OR** conditioned pain modulation.mp **OR** algometer.mp **OR** allodynia.mp **OR** hyperalgesia.mp

**AND**

Randomi* controlled trial.mp **OR** Non-randomi* controlled trial.mp **OR** Control trial.mp **OR** Intervention.mp **OR** Clinical trial*.mp **OR** comparison group.mp **OR** control group.mp **OR** non-random.mp

**PEDro**

Exercis* AND “Neck pain” AND threshold*

**Filters:**

Problem: Pain

Body Part: Head or neck

Topic: Chronic pain

Method: Clinical trial

**PubMed**

Exercis*[tw] **OR** training **OR** exercise training[tw] **OR** motor control[tw] **OR** physical activit*[tw] **OR** physical exercise[tw] **OR** resistance[tw] **OR** strength[tw] **OR** endurance[tw] **OR** exercise[tw] **OR** stretching[tw] **OR** pilates[tw] **OR** Core stab* **OR** yoga

**AND**

Neck pain[tw] **OR** neck injur*[tw] **OR** neckache[tw] **OR** neck trauma[tw] **OR** cervical pain **OR** cervical injur*[tw] **OR** cervical trauma **OR** cervicodynia[tw] **OR** cervicalgia **OR** chronic non-specific neck pain[tw] **OR** chronic neck pain[tw] **OR** chronic nonspecific neck pain[tw] **OR** nonspecific neck pain[tw] **OR** whiplash[tw] **OR** whiplash injur*[tw]

**AND**

Quantitative sensory testing[tw] **OR** sensory thresholds/ **OR** pain thresholds/ **OR** threshold*[tw] **OR** detection threshold[tw] **OR** thermal[tw] **OR** QST[tw] **OR** pain sensitisation[tw] **OR** pain sensitization[tw] **OR** pain modulation[tw] **OR** pain tolerance[tw] **OR** temporal summation[tw] **OR** conditioned pain modulation[tw] **OR** algometer[tw] **OR** allodynia[tw] **OR** hyperalgesia[tw]

**AND**

Randomi* controlled trial[tw] **OR** Non-randomi* controlled trial[tw] **OR** Control trial[tw] **OR** Intervention[tw] **OR** Clinical trial*[tw] **OR** comparison group[tw] **OR** control group[tw] **OR** non-random[tw]

**Scopus**

Exercis* **OR** training **OR** “exercise training” **OR** “motor control” **OR** “physical activit*” **OR** “physical exercise” **OR** resistance **OR** strength **OR** endurance **OR** exercise **OR** stretching **OR** pilates **OR** “Core stab*” **OR** yoga

**AND**

“Neck pain” **OR** “neck injur*” **OR** neckache **OR** “neck trauma” **OR** “cervical pain” **OR** “cervical injur*” **OR “**cervical trauma” **OR** cervicodynia **OR** cervicalgia **OR “**chronic non-specific neck pain” **OR “**chronic neck pain” **OR** “chronic nonspecific neck pain” **OR** “nonspecific neck pain” **OR** whiplash **OR “**whiplash injur*”

**AND**

“Quantitative sensory testing” **OR “**sensory thresholds” **OR** “pain thresholds” **OR** threshold* **OR** “detection threshold” **OR** thermal **OR** QST **OR** “pain sensitisation” **OR** “pain sensitization” **OR** “pain modulation” **OR** “pain tolerance” **OR** “temporal summation” **OR** “conditioned pain modulation” **OR** algometer **OR** [allodynia](https://www.sciencedirect.com/topics/medicine-and-dentistry/allodynia) **OR** hyperalgesia

**AND**

“Randomi* controlled trial” **OR** “Non-randomi* controlled trial” **OR** “Control trial” **OR** Intervention **OR** “Clinical trial*” **OR** “comparison group” **OR** “control group” **OR** non-random

**Web of Science**

Exercis* **OR** training **OR** “exercise training” **OR** “motor control” **OR** “physical activit*” **OR** “physical exercise” **OR** resistance **OR** strength **OR** endurance **OR** exercise **OR** stretching **OR** pilates **OR** “Core stab*” **OR** yoga

**AND**

“Neck pain” **OR** “neck injur*” **OR** neckache **OR** “neck trauma” **OR** “cervical pain” **OR** “cervical injur*” **OR “**cervical trauma” **OR** cervicodynia **OR** cervicalgia **OR “**chronic non-specific neck pain” **OR “**chronic neck pain” **OR** “chronic nonspecific neck pain” **OR** “nonspecific neck pain” **OR** whiplash **OR “**whiplash injur*”

**AND**

“Quantitative sensory testing” **OR “**sensory thresholds” **OR** “pain thresholds” **OR** threshold* **OR** “detection threshold” **OR** thermal **OR** QST **OR** “pain sensitisation” **OR** “pain sensitization” **OR** “pain modulation” **OR** “pain tolerance” **OR** “temporal summation” **OR** “conditioned pain modulation” **OR** algometer **OR** [allodynia](https://www.sciencedirect.com/topics/medicine-and-dentistry/allodynia) **OR** hyperalgesia

**AND**

“Randomi* controlled trial” **OR** “Non-randomi* controlled trial” **OR** “Control trial” **OR** Intervention **OR** “Clinical trial*” **OR** “comparison group” **OR** “control group” **OR** non-random

**Google Scholar**

Exercis* AND “Neck pain” AND threshold*

References downloaded from first 10 pages (100)

**clinicaltrials.gov**

(Exercise OR training OR "exercise training" OR "motor control" OR "physical activity" OR "physical exercise" OR resistance OR strength OR endurance OR exercise OR stretching OR pilates OR "Core stability" OR yoga) AND ("Neck pain" OR "neck injury" OR neckache OR "neck trauma" OR "cervical pain" OR "cervical injury" OR "cervical trauma" OR cervicodynia OR cervicalgia OR "chronic non-specific neck pain" OR "chronic neck pain" OR "chronic nonspecific neck pain" OR "nonspecific neck pain" OR whiplash OR "whiplash injury") AND ("Quantitative sensory testing" OR "sensory thresholds" OR "pain thresholds" OR threshold OR "detection threshold" OR thermal OR QST OR "pain sensitisation" OR "pain sensitization" OR "pain modulation" OR "pain tolerance" OR "temporal summation" OR "conditioned pain modulation" OR algometer OR allodynia OR hyperalgesia) AND ("Randomised controlled trial" OR "Randomized controlled trial" OR "Non-randomised controlled trial" OR "Non-randomized controlled trial" OR "Control trial" OR Intervention OR "Clinical trial" OR "comparison group" OR "control group" OR non-random)

Filters applied:

Status: Completed

Age: Adult (18-64) AND Older adult (65+)

Study results: With results

Actual search:

3 Studies found for: ( Exercise OR training OR EXPAND[Concept] "exercise training" OR EXPAND[Concept] "motor control" OR EXPAND[Concept] "physical activity" OR EXPAND[Concept] "physical exercise" OR resistance OR strength OR endurance OR exercise OR stretching OR pilates OR EXPAND[Concept] "Core stability" OR yoga ) AND ( EXPAND[Concept] "Neck pain" OR EXPAND[Concept] "neck injury" OR neckache OR EXPAND[Concept] "neck trauma" OR EXPAND[Concept] "cervical pain" OR EXPAND[Concept] "cervical injury" OR EXPAND[Concept] "cervical trauma" OR cervicodynia OR cervicalgia OR EXPAND[Concept] "chronic non-specific neck pain" OR EXPAND[Concept] "chronic neck pain" OR EXPAND[Concept] "chronic nonspecific neck pain" OR EXPAND[Concept] "nonspecific neck pain" OR whiplash OR EXPAND[Concept] "whiplash injury" ) AND ( EXPAND[Concept] "Quantitative sensory testing" OR EXPAND[Concept] "sensory thresholds" OR EXPAND[Concept] "pain thresholds" OR threshold OR EXPAND[Concept] "detection threshold" OR thermal OR QST OR EXPAND[Concept] "pain sensitisation" OR EXPAND[Concept] "pain sensitization" OR EXPAND[Concept] "pain modulation" OR EXPAND[Concept] "pain tolerance" OR EXPAND[Concept] "temporal summation" OR EXPAND[Concept] "conditioned pain modulation" OR algometer OR allodynia OR hyperalgesia ) AND ( EXPAND[Concept] "Randomised controlled trial" OR EXPAND[Concept] "Randomized controlled trial" OR EXPAND[Concept] "Non-randomised controlled trial" OR EXPAND[Concept] "Non-randomized controlled trial" OR EXPAND[Concept] "Control trial" OR Intervention OR EXPAND[Concept] "Clinical trial" OR EXPAND[Concept] "comparison group" OR EXPAND[Concept] "control group" OR non-random ) | Completed Studies | Studies With Results | Adult, Older Adult

**ISRCTN registry**

(Exercise OR training OR "exercise training" OR "motor control" OR "physical activity" OR "physical exercise" OR resistance OR strength OR endurance OR exercise OR stretching OR pilates OR "Core stability" OR yoga) AND ("Neck pain" OR "neck injury" OR neckache OR "neck trauma" OR "cervical pain" OR "cervical injury" OR "cervical trauma" OR cervicodynia OR cervicalgia OR "chronic non-specific neck pain" OR "chronic neck pain" OR "chronic nonspecific neck pain" OR "nonspecific neck pain" OR whiplash OR "whiplash injury") AND ("Quantitative sensory testing" OR "sensory thresholds" OR "pain thresholds" OR threshold OR "detection threshold" OR thermal OR QST OR "pain sensitisation" OR "pain sensitization" OR "pain modulation" OR "pain tolerance" OR "temporal summation" OR "conditioned pain modulation" OR algometer OR allodynia OR hyperalgesia)

**Filters:**

**Trial Status:** Completed

**Participant age range:** Adult

**Results:** With results
