# Supplementary material for: The effects of neck exercise in comparison to passive or no intervention on quantitative sensory testing measurements in adults with chronic neck pain: A systematic review
Source: PLoS One. 2024 May 3;19(5):e0303166. doi: 10.1371/journal.pone.0303166 (PMC11068209; doi:10.1371/journal.pone.0303166)
Supplement: S4 File — (DOCX) [file pone.0303166.s004.docx]

**Supp File 4. Summary of Interventions**

| **Study** | **Summary of Intervention and Grouping** |
| --- | --- |
| Bernal-Utrera et al^[45]^ | 1. **Progressive Resistance Training**   Protocol performed once daily for 3 weeks (21 total sessions) based on introducing progressive load throughout different phases. Each phase correlates to 1 week of training.  ***Phase 1: Deep cervical flexor activation and recruitment***   - Cranio-cervical flexion in supine for 3 sets of 10 repetitions with 10 second hold for each repetition and 10 second rest. - Cranio-cervical flexion in sitting for 3 sets of 10 repetitions with 10 second hold for each repetition and 10 second rest.   ***Phase 2: Deep and superficial cervical flexor co-contraction isometric exercise***   - All exercises from Phase 1. - Deep and superficial cervical flexor co-contraction in supine decubitus with 10 second hold for 10 repetitions with 10 second rest. - Cranio-cervical flexion with ipsilateral rotation and lateral flexion, with contralateral resistance externally applied with 10 second hold for 10 repetitions with 10 second rest.   ***Phase 3: Eccentric recruitment of cervical flexors and extensors***   - All exercises from Phases 1 and 2. - Cervical extension to cranio-cervical flexion to cervical flexion in sitting for 10 repetitions to cause cervical extensor eccentric contraction. - Completed in 4-point kneeling position. Cervical flexion to cranio-cervical extension. Whilst maintaining cranio-cervical flexion, add neck extension and release cranio-cervical extension. Completed for 10 repetitions. |

| **Study** | **Summary of Intervention and Grouping** |
| --- | --- |
| Li et al^[46]^ | 1. **Progressive Resistance Training**   Protocol performed ≥3 times per week for 6 weeks. 10-minute warm-up consisting of cervical flexion, extension, lateral flexion, rotation, trapezius shrugs and deltoid lateral raises, performed before each session. Cervical exercise protocol performed in sitting with cervical spine in anatomical neutral position, with Theraband positioned around forehead and anchored to wall scale or handheld scale. Protocol began at 30% of participant’s maximal strength, progressing to 50% at week 2 and 70% at week 4, with progression achieved by utilising Theraband of greater resistance. Protocol consisted of cervical flexion, extension, left lateral flexion and right lateral flexion performed for 8-12 repetitions, with 5 second duration for each repetition. Participants also provided with booklet regarding principles of workplace ergonomics and a training diary to record adherence.   1. **Fixed Resistance Training**   Protocol the same as progressive resistance training for this study, but resistance fixed at 70% of participant’s maximal strength as evaluated at baseline assessment. Participants also provided with booklet regarding principles of workplace ergonomics and a training diary to record adherence. |
| Ris et al^[47]^ | 1. **Progressive Resistance Training Combined with Graded Physical Training**   Individualised programme consisting of cervical flexor and extensor function exercise, standing balance, oculomotor exercise and shoulder girdle neuromuscular function exercise, performed twice daily, 3 times per week, for four months.  In addition to the exercise protocol, participants completed a self-selected graded physical training programme, consisting of modalities such as cycling or walking. Starting duration for physical training was set 20% below the participant’s initial capacity indication, and progressed by 20% every 2 weeks, with training aimed to achieve a rating of perceived exertion between 11 and 14 on a 6-20 Borg Scale (Borg, 1990).  Participants additionally completed 4 sessions of pain management advice and education and 8 sessions of exercise and physical training instruction lasting 30 minutes per session. |

**Appendix E: Secondary Outcomes of Interest Data**

| **Study** | **Outcome Measure** | **Key Findings** |
| --- | --- | --- |
| Bernal-Utrera et al^[45]^ | Visual Analogue Scale | Statistically significant improvement noted between groups for progressive resistance training in comparison to control at week 4 (*p*=0.001) and week 12 (*p*=0.007).  A medium effect size was noted for progressive resistance training in comparison to control at week 2 (r^2^=0.113) and a large effect size at week 4 (r^2^=0.496) and week 12 (r^2^=0.209). |
|  | Neck Disability Index | Statistically significant improvement noted between groups for progressive resistance training in comparison to control at weeks 2 (*p*=0.001), 4 (*p*=0.001) and 12 (*p*=0.001).  A large effect size was noted for progressive resistance training in comparison to control at week 2 (r^2^=0.430), week 4 (r^2^=0.629) and week 12 (r^2^=0.459). |
| Li et al^[46]^ | Visual Analogue Scale | **Fixed Resistance Training**  Statistically significant improvement noted between groups for fixed resistance training in comparison to control at week 4 (*p*=0.012), week 6 (*p<*0.001) and 3 months (*p<*0.001).  **Progressive Resistance Training**  Statistically significant improvement noted between groups for progressive resistance training in comparison to control at week 4 (*p<*0.001), week 6 (*p<*0.001) and 3 months (*p<*0.001). |

| **Study** | **Outcome Measure** | **Key Findings** |
| --- | --- | --- |
| Li et al^[46]^ continued | Neck Disability Index | **Fixed Resistance Training**  Statistically significant improvement noted between groups for fixed resistance training in comparison to control at week 4 (*p*=0.036), week 6 (*p<*0.001) and 3 months (*p<*0.001).  **Progressive Resistance Training**  Statistically significant improvement noted between groups for progressive resistance training in comparison to control at week 4 (*p<*0.001), week 6 (*p<*0.001) and 3 months (*p<*0.001). |
| Ris et al^[47]^ | Pain Bothersomeness Scale | No statistically significant improvement noted between groups for progressive resistance training combined with graded physical training in comparison to control at 4-month follow-up (*p*=0.68). |
|  | Neck Disability Index | No statistically significant improvement noted between groups for progressive resistance training combined with graded physical training in comparison to control at 4-month follow-up (*p*=0.62). |
